# The Toothbrushing Effects on Surface Properties and Color Stability of CAD/CAM and Pressable Ceramic Fixed Restorations—An In Vitro Study

**DOI:** 10.3390/ma16082950

**Published:** 2023-04-07

**Authors:** Amr A. Mahrous, Abdullah Alhammad, Faisal Alqahtani, Yousif Aljar, Ahmed Alkadi, Noha Taymour, Abdulkareem Alotaibi, Sultan Akhtar, Mohammed M. Gad

**Affiliations:** 1 Department of Substitutive Dental Sciences, College of Dentistry, Imam Abdulrahman Bin Faisal University, P.O. Box 1982, Dammam 31441, Saudi Arabia; mganem@iau.edu.sa (A.A.M.); ntyoussef@iau.edu.sa (N.T.); amoalotaibi@iau.edu.sa (A.A.); 2 College of Dentistry, Imam Abdulrahman Bin Faisal University, P.O. Box 1982, Dammam 31441, Saudi Arabia; 2160004639@iau.edu.sa (A.A.); 2160006256@iau.edu.sa (F.A.); 2160001812@iau.edu.sa (Y.A.);; 3 Department of Biophysics, Institute for Research and Medical Consultations (IRMC), Imam Abdulrahman Bin Faisal University, P.O. Box 1982, Dammam 31441, Saudi Arabia; suakhtar@iau.edu.sa

**Keywords:** ceramic material, CAD/CAM, color change, fixed dental prostheses, surface properties

## Abstract

Pressable ceramic restorations have been introduced and investigated, and found comparable to CAD/CAM ceramic in terms of mechanical properties; however, the effect of toothbrushing on the pressable ceramic has not been thoroughly investigated. The objective of the current study was to assess the effect of artificial toothbrushing simulation on the surface roughness, microhardness, and color stability of different ceramic materials. Three lithium disilicate-based ceramics (IPS Emax CAD [EC], IPS Emax Press [EP]; (Ivoclar Vivadent AG), and LiSi Press [LP] (GC Corp, Tokyo, Japan)) were examined. For each ceramic material, eight bar-shaped specimens were prepared and subjected to 10,000 brushing cycles. Surface roughness, microhardness, and color stability (∆E) were measured before and after brushing. Scanning electron microscopy (SEM) was used for surface profile analysis. The results were analyzed using one-way ANOVA, Tukey’s post hoc test, and paired sample *t*-test α = 0.05. The findings revealed a non-significant decrease in the surface roughness of EC, EP, and LP groups (*p* > 0.05), and both LP and EP have the lowest surface roughness values (0.64 ± 0.13, 0.64 ± 0.08 µm) after brushing, respectively. Toothbrushing showed a decrease in the microhardness of the three groups: EC and LP, *p* < 0.001; EP, *p* = 0.012). EP showed the lowest hardness value after brushing (862.45 ± 273.83). No significant changes (∆E) were observed in all groups (*p* > 0.05); however, the EC group was found to be considerably affected by color changes, in comparison to the EC and LP groups. Toothbrushing had no effect on surface roughness and color stability of all tested materials, but it decreased the microhardness. Material type, surface treatments, and glazing of ceramic materials contributed to the surface changes in the ceramic materials, necessitating further investigations in terms of the toothbrushing effect with different glazing as variables.

## 1. Introduction

Dental ceramics are inorganic, non-metallic materials used as dental prostheses to replace missing teeth and restore defective dental structures. Ceramics are the material of choice for long-term esthetics and stability [1]. Ceramics are classified into three categories based on their microstructure: polycrystalline, glass-based ceramics, and crystalline fillers [2]. All-ceramic restorations have become popular because they mimic the appearance of natural teeth [3]. Moreover, it is affected by different factors, including the type of material used, manufacturing process, prosthesis design, and the oral environment [4,5].

Many techniques have been reported for fabricating dental ceramic restorations. The conventional layering technique was designed to be used with the feldspathic porcelain, where the porcelain particles are mixed with glycerin or distilled water and are to be added in layers, mainly for metal-ceramic restorations. The limitations of this technique are that it is time-consuming, technique sensitive, and unpredictable [6]. Ceramic core materials, such as lithium disilicate, zirconium oxide, and aluminum oxide, have their own systems, manufacturing techniques, and clinical indications [7]. Due to different ceramic surface finishing protocols, surface roughness, topography, and surface light scattering could be affected. A certain protocol should be followed to prevent damage to the optical properties of the ceramic material [8]. Many products in the market showed a significant improvement in polishing capabilities. However, the shade changes that occur with abrasion and the ability to recover shade changes after using the polishing systems still need more improvements [9]. A previous study by Manjuran et al. [10], investigating different ceramic specimens, showed that a smoother porcelain surface could be obtained after polishing using ceramics without a notable effect on color. Recently, there has been an increasing concern in the study of the factors affecting the surface characteristics of ceramic materials. Superficial changes of ceramic materials may be provoked by chemical aging and diet, which could be simulated by immersion in food-simulating liquids (FSLs), and mechanical aging by toothbrushing [11].

Toothbrushing, as an example of surface wear, is another main factor that might influence the surface properties of ceramic materials. Many studies [12,13,14,15] have reported that toothbrushing can adversely affect feldspathic porcelain restorations [12]. A previous study reported that extrinsically stained feldspathic porcelain restorations showed significant alteration in surface roughness and color after 8.5 years of simulated toothbrushing [13]. Dal Piva et al. reported that using a toothbrush could remove extrinsic stains applied onto the surface of pressable ceramics after 10 to 12 years of toothbrushing unless the stain is covered by a protective layer of glaze [14]. Other studies showed no effect of artificial toothbrushing simulation on surface roughness, microhardness, or the color stability of lithium disilicate ceramics after five simulated years [15].

The study of the effect of toothbrushing on restorations can lead to the development of new materials and techniques that are more durable and stable, improving the quality and longevity of dental restorations, as well as assisting in the selection of appropriate materials and techniques for fabrication. To our best knowledge, few studies have examined the influence of toothbrushing on different pressable and machined lithium disilicate ceramics. Therefore, this current study was conducted to assess and compare the effect of toothbrushing on the surface roughness, microhardness, and color stability of different ceramic restorations. The null hypothesis stated that toothbrushing has no significant effect on the surface roughness, microhardness, and color stability of tested ceramic materials.

## 2. Materials and Methods

G*Power ((Version 3.1.9.4) 5% alpha error, effect size = 0.59) was used for the sample size calculation [16]. The calculation revealed that the minimum sample size was 9 per test group, which was increased to 10 specimens to counteract any testing errors. A total of 30 specimens were included. The specimens were prepared with a standardized dimension of 16 (length) × 6 (width) × 1.2 (height) mm^3^. The materials’ information, compositions, and fabrications are summarized in Table 1.

### 2.1. Specimens’ Finishing and Polishing

As per the manufacturer’s recommendations, all the ceramic specimens were prepared and cut by using a diamond saw (ISOMET 1000, Buehler, Lake Bluff, IL, USA). This was on a low-speed under-water cooling system until the desired bar-shaped specimens with dimensions of 16.0 × 6.0 × 1.2 mm^3^ were obtained, as per the International Organization of Standards (ISO standards 178:2010) [17]. Finishing and polishing for specimens are summarized in Table 1 according to previous studies [14,18].

### 2.2. Toothbrushing Protocol

The protocol of toothbrushing was performed by subjecting the specimens to an electric toothbrushing simulation unit (model ZM-3.8, SD Mechatronik, Feldkirchen-Westerham, Germany) for 10,000 cycles, which is equivalent to 1 year of clinical toothbrushing simulation [14,19]. Brush type (Oral-B PRO 1000, Leicester, UK) and Colgate toothpaste slurry solution (250 g with 1 L of distilled water) were replaced after 5000 cycles [14]. Direction and pressure were standardized by fixing the electric brush on a prefabricated stainless steel holder. The Cross Action brush head is aligned with the bristles, with a vertical load of 2.45 N and 180 strokes per minute as the brushing rate.

### 2.3. Surface Roughness Test (Ra)

A non-contact profilometer (Contour GT-K1 optical profiler; Bruker Nano, Tucson, AZ, USA) was used to measure Ra before and after toothbrushing. A custom Putty holder was prepared to standardize Ra measuring before and after brushing, and fixed points were marked on the sides. Three points were scanned per specimen, and the average (Ra) per specimen was recorded [17].

### 2.4. Scanning Electron Microscopy (SEM) Analysis

Representative specimens from each ceramic group before (EC-A, EP-A, and LP-A) and after toothbrushing (EC-B, EP-B, and LP-B) were ultrasonically cleaned using distilled water and sputter coated with gold. They were then analyzed using scanning electron microscopy (SEM) (INSPECT S50, FEI, Brno, Czech Republic; 20 kV) to determine the surface topography and surface features [17]. The SEM images were captured at different magnifications; however, SEM images of each specimen were displayed at a representative magnification of ×2000.

### 2.5. Microhardness Test

Specimens used in this study were examined before and after toothbrushing to measure surface microhardness. A Vickers hardness testing device (Wilson Hardness; ITW Test & Measurement GmbH, Shanghai, China) was used to measure the microhardness. Via the indenter, a loading (100 gf, dwell time of 15 s) was applied at three different points per specimen [17], and the average of the three points was calculated.

### 2.6. Color Stability (ΔE)

The digital spectrophotometer (Color-Eye 7000A; X-rite, Grand Rapids, MI, USA) was used to capture the color changes by differentiating the transmitted and reflected light beam for each specimen using CIE L*a*b* [20]. To analyze the color stability of each specimen, the following equation was used: ∆E=[∆L(L−L*)2 +∆a(a−a*)2+∆b(b−b*)2]12 [21] (color changes (∆E) and color variables (L*, a*, b*). To apply the color changes in this research in a clinical situation, a formula was used to convert the color changes (∆E) to National Bureau of Standards (NBS) units (NBS units = E × 0.92). The formula used to classify the clinically acceptable color changes is as follows: indicial (NBS = 0.0–0.5); slight (NBS = 0.5–1.5); noticeable (NBS = 1.5–3.0); considerable (NBS = 3.0–6.0); very (NBS = 6.0–12.0); and excessive (NBS = +12.0) [22].

### 2.7. Statistical Analysis

Numerical data based on measurements of Ra, microhardness, and color change were tabulated as mean and standard deviation (SD). A normality test (Kolmogorov–Smirnov test) showed normal data distribution for Ra and microhardness. A one-way ANOVA was used to compare the tested properties between the different study groups and post hoc Tukey’s test. A paired sample *t*-test was also used to compare mean differences before and after toothbrushing. A non-Gaussian distribution of color change (ΔE) data was found, so a non-parametric Kruskal–Wallis test was applied as an alternative to the ANOVA test. A post hoc Mann–Whitney U test was performed. A statistically significant difference was set at *p*-value ≤ 0.05. SPSS v.22.0 (IBM product, Chicago, IL, USA) was used for data analysis.

## 3. Results

Mean surface roughness was compared between the materials using a one-way ANOVA that revealed a non-significant difference in means before toothbrushing (F = 0.007, *p* = 0.993) and after toothbrushing (F = 1.74, *p* = 0.842). The paired differences of means within the study group for comparison of mean surface roughness before and after toothbrushing were also non-significant in each group, at a 5% significance level (Table 2).

**Figure 1:**
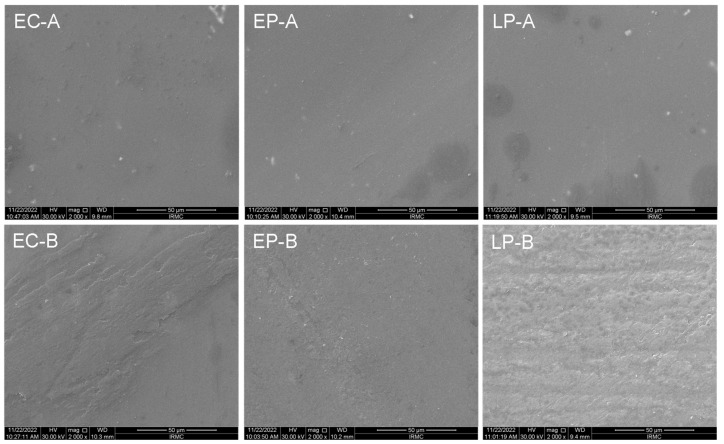
SEM images of the CAD/CAM specimens before (EC-A, EP-A, and LP-A) and after ceramic brushing (EC-B, EP-B, and LP-B). SEM magnification: ×2000.

Surface topography examined under SEM revealed different surface roughness behavior for EC, EP, and LP groups before toothbrushing. However, the LP group showed a higher surface roughness, as shown in Figure 1. The surface roughness profiles have been evaluated after simulated toothbrushing for each ceramic material, and SEM images showed a smooth surface of glazed specimens for all tested materials, although the EC group showed surface micro-irregularities.

Mean microhardness was compared between the materials using a one-way ANOVA that revealed a non-significant difference of means before toothbrushing (F = 0.567, *p* = 0.576), though the difference was significant after toothbrushing (F = 3.66, *p* = 0.043). The paired differences of means within the study group for comparison of mean surface roughness before and after toothbrushing were found significant in each group at a 5% significance level (Table 2).

Analysis of primary color parameters (L*, a* and b*) were performed by employing one-way ANOVA between the materials. The results of ΔL* showed a significant difference of means between the materials before thermo-cycling (F = 8.36, *p* = 0.002) but non-significant after thermo-cycling (F = 0.85, *p* = 0.441). Paired differences of means ΔL* within materials EP and LP were non-significant (*p* > 0.05); however, it was significant in EC (*p* = 0.012). The results of mean Δa* between materials before and after toothbrushing were non-significant, respectively (F = 0.716, *p* = 0.500) and (F = 3.24, *p* = 0.060). Paired differences of means within each material revealed significance in EC (*p* = 0.012) and LP groups (*p* = 0.017). The results of mean Δb* between materials before toothbrushing (F = 167.7, *p* < 0.001) and after toothbrushing (F = 23.45, *p* < 0.001) were found highly significant. However, the paired differences of means of Δb* within each material before and after toothbrushing were non-significant (Table 3).

The mean color change ΔE in EP, EC, and LP was 4.2 ± 1.55, 7.86 ± 4.13, and 11.67 ± 3.20, respectively, and was found to be non-significant when employing the Kruskal–Wallis test following a non-Gaussian distribution (F = 1.27, *p* = 0.299). A post hoc Mann–Whitney U-test for pairwise comparisons of color change (ΔE) between the materials is presented in Table 4. However, EC showed considerable color change when compared with EP and LP, which showed no detectable changes, as shown in Table 5.

## 4. Discussion

The impact of artificial toothbrushing on the color change, surface roughness (Ra), and microhardness of CAD/CAM and pressable lithium disilicate dental ceramic materials was examined. The null hypothesis of this current study was rejected for the EC, EP, and LP groups for surface microhardness; however, the null hypothesis was not rejected for the surface roughness and color changes for all groups. With the increased demand for monolithic restorations, ceramic materials with various compositions have evolved to be applied in monolithic restorations, mainly for mimicking natural tooth shade and achieving comparable strength [23]. IPS Emax CAD and IPS Emax Press are widely used in the dentistry of fixed restorations, which include crowns and fixed partial dentures for anterior teeth [24]. LiSi Press was recently introduced into the dental market with minimal research and testing [25].

One of the various laboratory aging protocols is toothbrushing simulation [26], which simulates the clinical condition where abrasion could affect the esthetics and longevity of ceramic restorations. Hence, subjecting specimens to such wear after simulated toothbrushing can be used as a guide for the clinical performance of tested materials. Most of the abrasive simulations utilizing toothbrushing evaluated wear rate instead of surface roughness quality [27,28]. In addition, the polishing surface is subjected to oral conditions and could be influenced by long-term toothbrushing [29]. However, limited data are available concerning the influence of toothbrushing on machinable and pressable ceramics over one year.

The current study aimed to assess the impact of toothbrushing simulation on different CAD/CAM and pressable lithium disilicate ceramics in terms of surface roughness, microhardness, and color change. Based on our findings, the Emax CAD, Emax Press, and LiSi Press groups showed a decrease in the surface roughness (Ra) after an equivalent of 1 year of toothbrushing; however, the decrease was insignificant, which may be explained by their excellent physicomechanical stability [19,30]. Additionally, no significant difference in Ra values was observed among the groups after mechanical toothbrushing (*p* value = 0.842). This is in accordance with previous studies that showed a non-significant change in the roughness (Ra) of extrinsically stained pressable and CAD/CAM lithium disilicate ceramics after toothbrushing [15,31,32]. On the contrary, other studies showed an increased surface roughness of ceramic materials after different toothbrushing cycles. This inconsistency could be due to the differences in the load and type of toothbrush (e.g., use of a higher brushing load at 5.88 N and harder nylon toothbrushes compared to soft toothbrushes) [33,34].

SEM analysis showed a variation in surface roughness after brushing, as some faint serration lines started to appear, compared to the glazed specimens. However, it was observed that Ra values were non-significantly decreased for all materials. This may be attributed to the glaze effect the samples showed when received from the laboratory; glaze film may show peaks and valleys, resulting in a rougher surface. Toothbrushing affects these glazed materials and shows that the material substrates with smooth surfaces are related to the inherent surface properties of each material [35]. This finding focuses on the glaze type (e.g., materials and method of applications as a variable for further investigation, as the glaze may affect ceramic surface properties with long-term effects with different aging conditions) [36]. Despite using a non-vacuum firing cycle for the overglaze layer, the surface exhibited a few ruptured air bubbles on the surface. This toothbrushing simulation for the specimens partially denuded the superficial pitted surface of the glaze, exposing the more dense layer with fewer air bubble inclusions. This could explain the SEM results.

This finding supports the findings made by Meng et al., who reported that different aging conditions did not influence the surface roughness of lithium disilicate glass-ceramic [37]. Moreover, Goudas et al. examined the surface roughness of pressable dental ceramics after different aging conditions and reported that lower surface roughness values were observed [38]. On the contrary, previous studies showed that toothbrushing significantly increases surface roughness [13,39].

Surface hardness is an important property that compares dental materials’ resistance to surface penetration through the effect of brittle fractures and plastic flow, collectively. It can identify the wear resistance of materials, in addition to their abrasion effect on the opposing materials, which can directly influence materials’ finishing and polishability attributes [40]. Microhardness results showed no significant difference between the tested groups before toothbrushing procedures: however, a significant difference was found between the tested ceramics after toothbrushing, which might be associated with the diversity of microstructures that could be affected by the toothbrushing procedure, as well as the abrasive particles in the tooth paste, which can cause microcracks and scratches on the surface of the ceramic materials, and, subsequently, can decrease its microhardness. Several aspects should be considered to better determine ceramics’ hardness data, relating to surface properties, sizes, and boundaries of grain and crystalline phases [41] and oxidative stresses [42].

Moreover, another reason behind the significant decrease in the microhardness of EC, EP, and LP after a simulation of 1 year of toothbrushing is the glaze on the ceramic surface [15,43]. The findings of this study are in accordance with Rodrigues et al. [44], who reported that toothbrushing negatively affects glazed ceramic materials, which is concurrent with SEM findings. However, regarding the Emax Press group, the findings of this research do not coincide with previous data, which reported that the pressable ceramics showed higher microhardness than CAD/CAM ceramics [45,46,47]. Although it was found that the microhardness of the LP group was significantly higher than the EC and EP groups after toothbrushing, this may be related to the newly introduced pressable ceramic material offering higher thermal and shock resistance. In addition, the lithium disilicate glass-ceramic pressing technique undergoes a considerable plastic deformation that can align the crystals in a parallel direction to the pressing direction, hindering the microcracks’ propagation; this could be the reason behind the increased microhardness of the LP group [45,46,47].

Esthetics is one of the essential aspects to be considered in the selection of a restorative material. The CAD/CAM and pressable lithium disilicate ceramics investigated in the current study are clinically used to construct anterior or posterior fixed restorations. Recent data have shown their excellent optical characteristics [48]. These materials allow clinical reliability [49] and facilitate good patient feedback [50]. The optical characteristics of dental ceramic materials are influenced by the processing method, inherent microstructure, chemical structure, and physical characteristics of polycrystalline ceramics, such as the content and size of crystals, homogeneity, refractive index, and surface microporosity [48]. Color changes can be evaluated either conventionally, or by an instrumental technique. The spectrophotometer is one color-measuring instrument that can reduce human errors and provide a more objective match in detecting color change [51]. It is necessary to have acceptable reference values for evaluating color change results. Color changes are said to be clinically acceptable when ΔE > 1.045 and clinically unacceptable when ΔE > 3.5 [52]. In the present study, after artificial toothbrushing, a non-significant color alteration (ΔE) was detected for the Emax CAD, Emax Press, and LiSi Press. However, the color changes of the tested materials are unacceptable in comparison to the recommended references (E > 3.5). This could be explained by the presence of glaze as a protective layer applied over the ceramic materials, and the proper polishing protocol could protect the ceramic material against mechanical toothbrushing, comparable to the effect of glazing [33]. Moreover, non-significant color change (ΔE) was observed when comparing the Emax CAD and Emax Press groups, as well as between LISI Press Group, Emax CAD, and Emax Press individually; this might be due to the fact that the toothpaste used for toothbrushing is not likely to cause chemical changes to the material, which could also contribute to color alteration [28].

These findings coincide with Yuan et al., who found no significant color changes between CAD/CAM lithium disilicate ceramics after simulated toothbrushing [19]. Additionally, Garza et al. found that no significant difference was detected for shade change over time, irrespective of the technique for the lithium disilicate fabrication [53]. However, Schelkopf et al. examined the effect of toothbrushing on the color stability of lithium disilicate ceramics. They noticed that a significant color change was detected in comparison with the baseline, which might be due to their testing polished ceramic surfaces without the application of a glaze layer, as performed in this study [54].

Regarding our findings on Emax CAD, the change in the shade was higher than the acceptable threshold (NBS = 3.89). Still, it was the lowest, compared with Emax Press (NBS = 7.23) and LiSi Press (NBS = 10.73), and as such might be advocated for its improved clinical color stability. Another reason is the difference in the firing temperatures, which might affect the glaze layer on the Emax CAD [15,55]. These findings are in accordance with Palla et al. [55], who reported a noticeable color change was found in CAD/CAM lithium disilicate specimens, although this difference was not significant. Furthermore, Anil et al. [31] investigated the effect of various aging conditions on the color stability of pressable ceramics and found that the color change was clinically acceptable. Additionally, Maciel et al. found that the color change within the pressable ceramics is within the clinically acceptable range [52].

Our results contrast with a previous study, which showed that the ΔE values of the CAD/CAM ceramics increased, and these changes were clinically acceptable [51]. The variations between this study and previous ones are mainly due to using different finishing and polishing protocols.

It should be mentioned that while surface roughness is associated with ceramic color change, it is not the only reason for staining. One possible justification is that the pigmentation of ceramics is related to both extrinsic and intrinsic factors [52]. For example, the Emax Press and LiSi Press have a translucent structure, with various crystalline forms, compared to the other ceramics, and their reactivity contributes to their transparency, making the surface less prone to mechanical attrition by toothbrushing, and thus not affecting the shade of the material [56]. Furthermore, this obvious advantage becomes a drawback when pigment particles impregnate its surface, changing its luminosity and, as a result, negatively influencing the shade of the indirect restorations. Therefore, further laboratory investigations are needed in this regard. In addition, it was found from the results that the Emax CAD has a rougher surface than the other two pressable ceramics, and, consequently, the color change was more considerable in this group; however, the difference was non-significant. It is noteworthy to mention that the investigated materials are also influenced by the substrate shade that, together with color stability, determines the ultimate esthetic appearance of a ceramic restoration [57].

The current American Dental Association (ADA) recommendation is to replace the toothbrush every 3–4 months. This rate might modify the findings of the current study. In the current study, the toothbrushes were changed after 5000 simulated toothbrushing cycles [14,29] because of the experimental design and time management. In the case of a loss of stiffness of the toothbrush bristles used in the experiment, this might result in a slight increase in surface roughness. However, no direct comparisons could be applied with earlier studies because of the use of different protocols and dissimilar materials. Additional experimental studies will be necessary to examine the influence of various brushing systems, such as electronic toothbrushes and fluoride toothpaste.

Despite the limitations of the current study, tooth restorations using these ceramic materials can be recommended for permanent use in patients with moderate tooth wear. One of the limitations is that it was an in vitro laboratory experiment with a short simulated toothbrushing time, which might have limited effects on the ceramic surface. Increasing the toothbrushing cycle time could influence the clinical preference for pressable and CAD/CAM lithium disilicate ceramics materials. Furthermore, the ceramic specimens were glazed on both sides, which differs from the clinical condition, in which the ceramic restoration is bonded to the surface of the tooth. The influence of surface roughness on the color stability of the CAD/CAM ceramics should be investigated and related to the spectrophotometer recordings. Various data should be obtained with various types of porcelain and polishing techniques [58]. More experimental studies are required using various ceramic materials, thicknesses, pigmentation, and simulated toothbrushing cycles in addition to increasing the number of sample. Keeping in mind the possible clinical implications when using various dental ceramics and polishing protocols is important, which allows the possibility of finding outcomes that can sustain their indications.

## 5. Conclusions

Little evidence was reported on the influence of toothbrushing on the color stability and surface properties of pressable and machined lithium disilicate ceramics. Therefore, this current study was conducted to assess and compare the effect of toothbrushing on the surface roughness, microhardness, and color stability of different ceramic restorations. Based on the findings of present study, the following conclusions could be drawn:

The surface roughness of Emax CAD, Emax Press and LISI Press are relatively resistant to 1 year equivalent toothbrushing. Emax CAD, Emax Press and LISI Press showed a decrease in the microhardness after 1 year equivalent toothbrushing. Emax CAD, Emax Press and LiSi Press are relatively resistant to color change. However, the Emax CAD ceramics exhibited considerable color change values with an equivalent to 1 year of simulated toothbrushing, compared to the pressable ceramics (Emax Press and LiSi Press). Regardless of material type, the change in tested properties is mainly attributed to the glazing, which improves the surface properties, especially after surface adjustment; therefore, further investigations focusing on glazing type and methods are recommended.

## Figures and Tables

**Table 1:** Materials used in the present study (specification and manufacturing methods).

	Materials		
	Emax CAD (EC)	Emax Press (EP)	LiSi Press (LP)
**Brand name**	IPS Emax CAD, Ivoclar Vivadent, Schaan, Liechtenstein.	IPS Emax Press, Ivoclar Vivadent, Schaan, Liechtenstein.	GC Initial LiSi Press, GC Corp, Tokyo, Japan.
**Composition**	SiO_2_, Li_2_O, K_2_O, P_2_O_5_, ZrO_2_, ZnO, Al_2_O_3_, MgO.	SiO_2_, Li_2_O, K_2_O, P_2_O_5_, ZrO_2_, ZnO.	SiO_2_, Al_2_O_3_, Li_2_O, K_2_O, ZrO_2_, Na_2_O, P_2_O_5_, ZrO_2_.
**Fabrication techniques**	Diamond saw on low speed under water cooling (ISOMET 5000 Linear Precision Saw, Buehler Ltd., IL, USA).		
**Finishing and polishing**	Grit carbide sandpapers (#600, #1200, and #2400) under running water utilizing a polishing machine (MetaServ 250 Grinder-Polisher with Vector Power Head, Buehler, IL, USA).		
**Glazing**	All the ceramic materials were glazed as per the manufacturer’s instructions (710 °C, Programat CS2; Ivoclar Vivadent AG). Vacuum stages: St 1: 450. St 2: 709. Pre-heat: 6-min, then it will raise the temperature by 60 degrees each min until it reaches 710 degrees. After 1-min, start cooling.		

**Table 2:** Toothbrushing effects the surface characteristics of all tested materials.

Properties	Brushing	EC	EP	LP	^+^ P
**Surface roughness (µm)**	Before	0.73 ± 0.06	0.73 ± 0.02	0.77 ± 016	0.993
	After	0.67 ± 0.16	0.64 ± 0.13	0.64 ± 0.08	0.842
	^++^ p	0.327	0.069	0.161	
**microhardness (VHN)**	Before	1429.92 ± 144.25	1381.82 ± 101.31	1458.42 ± 179.86	0.576
	After	1119.51 ± 164.47	862.45 ± 273.83	1084.30 ± 159.72	0.043
	^++^ p	0.012	0.001	<0.001	

Non-significant difference of means between the groups at *p* ≤ 0.05 level. ^+^ One-way ANOVA, ^++^ paired sample *t*-test.

**Table 3:** The changes in color parameters (ΔL*, Δa*, Δb*) analysis in terms of brushing effect.

Color Parameters		Materials			^+^ P
		EC	EP	LP	
L*	Before	65.18 ± 2.60	68.74 ± 1.43	65.70 ± 1.35	0.002
	After	61.89 ± 5.16	60.95 ± 8.04	55.94 ± 14.01	0.441
	ΔL*	3.29 ± 5.54	7.79 ± 8.02	9.76 ± 14.44	
	^++^ P	0.093	0.012	0.123	
a*	Before	−1.91 ± 0.72	−1.72 ± 0.09	−1.67 ± 0.08	0.500
	After	−1.72 ± 0.62	−1.17 ± 0.45	−1.06 ± 0.58	0.060
	Δa*	−0.19 ± 0.36	−0.56 ± 0.46	−0.61 ± 0.61	
	^++^ P	0.208	0.012	0.017	
b*	Before	−1.13 ± 1.02	5.04 ± 0.62	4.93 ± 0.59	<0.001
	After	−0.68 ± 0.70	4.39 ± 1.11 ^a^	4.19 ± 2.60 ^a^	<0.001
	Δb*	−0.45 ± 0.90	0.64 ± 1.40	0.74 ± 2.55	
	^++^ P	0.123	0.123	0.575	

^+^ One-way ANOVA test for comparison between the materials and post hoc Tukey’s test. ^++^ Paired sample *t*-test for comparison between before and after toothbrushing results. ^a^. The mean difference is significant versus EC at *p* ≤ 0.05.

**Table 4:** Toothbrushing effect on the color change (ΔE) of all tested materials.

Color Change	EC	EP	LP	^+^ *p*-Value
**ΔE**	4.23 ± 1.55	7.86 ± 4.13	11.67 ± 3.02	0.251
**^++^ P**	---	Vs. EC = 0.195	Vs. EC = 0.161	
	---	--	Vs. EP = 0.878	

Non-significant difference of means between the groups at *p* ≤ 0.05 level. ^+^ Non-parametric Kruskal–Wallis test, ^++^ Mann–Whitney U test.

**Table 5:** ΔE in color changes in NBS values.

Materials	ΔE	NBS	Clinical Considerations
**EC**	4.23 ± 1.55	3.89	considerable (NBS = 3.0–6.0)
**EP**	7.86 ± 4.13	7.23	very (NBS = 6.0–12.0)
**LP**	11.67 ± 3.02	10.73	very (NBS = 6.0–12.0)

## Data Availability

The data are available upon request via email or phone to the corresponding author.
